# Prediction of overt hepatic encephalopathy after transjugular intrahepatic portosystemic shunt treatment: a cohort study

**DOI:** 10.1007/s12072-021-10188-5

**Published:** 2021-05-11

**Authors:** Yang Yang, Sirui Fu, Bin Cao, Kenan Hao, Yong Li, Jianwen Huang, Wenfeng Shi, Chongyang Duan, Xiao Bai, Kai Tang, Shirui Yang, Xiaofeng He, Ligong Lu

**Affiliations:** 1 Zhuhai Interventional Medical Centre, Zhuhai People’s Hospital (Zhuhai Hospital Affiliated With Jinan University), No. 79 Kangning Road, Zhuhai, 519000 Guangdong Province China; 2 Department of General Surgery, Zhuhai People’s Hospital (Zhuhai Hospital Affiliated With Jinan University), No. 79 Kangning Road, Zhuhai, China; 3 Division of Vascular and Interventional Radiology, Department of General Surgery, Nanfang Hospital, Southern Medical University, No. 1838 Guangzhou Avenue North, GuangzhouGuangdong Province, 510515 China; 4 Department of General Medicine, Zhuhai People’s Hospital (Zhuhai Hospital Affiliated With Jinan University), No. 79 Kangning Road, Zhuhai, China; 5 Department of Biostatistics, School of Public Health, Southern Medical University, No. 1838 Guangzhou Avenue North, Guangzhou, China; 6 Department of Nuclear Medicine, Zhuhai People’s Hospital (Zhuhai Hospital Affiliated With Jinan University), No. 79 Kangning Road, Zhuhai, China

**Keywords:** TIPS, Nervous system toxicity, Preoperative prediction, Combined model, Clinical factor, Imaging characteristics, Discrimination, Calibration, Decision curve, Risk stratification

## Abstract

**Background/purpose:**

Overt hepatic encephalopathy (HE) risk should be preoperatively predicted to identify patients suitable for curative transjugular intrahepatic portosystemic shunt (TIPS) instead of palliative treatments.

**Methods:**

A total of 185 patients who underwent TIPS procedure were randomised (130 in the training dataset and 55 in the validation dataset). Clinical factors and imaging characteristics were assessed. Three different models were established by logistic regression analyses based on clinical factors (Model^C^), imaging characteristics (Model^I^), and a combination of both (Model^CI^). Their discrimination, calibration, and decision curves were compared, to identify the best model. Subgroup analysis was performed for the best model.

**Results:**

Model^CI^, which contained two clinical factors and two imaging characteristics, was identified as the best model. The areas under the curve of Model^C^, Model^I^, and Model^CI^ were 0.870, 0.963, and 0.978 for the training dataset and 0.831, 0.971, and 0.969 for the validation dataset. The combined model outperformed the clinical and imaging models in terms of calibration and decision curves. The performance of Model^CI^ was not influenced by total bilirubin, Child–Pugh stages, model of end-stage liver disease score, or ammonia. The subgroup with a risk score ≥ 0.88 exhibited a higher proportion of overt HE (training dataset: 13.3% vs. 97.4%, *p* < 0.001; validation dataset: 0.0% vs. 87.5%, *p* < 0.001).

**Conclusion:**

Our combination model can successfully predict the risk of overt HE post-TIPS. For the low-risk subgroup, TIPS can be performed safely; however, for the high-risk subgroup, it should be considered more carefully.

**Graphic abstract:**

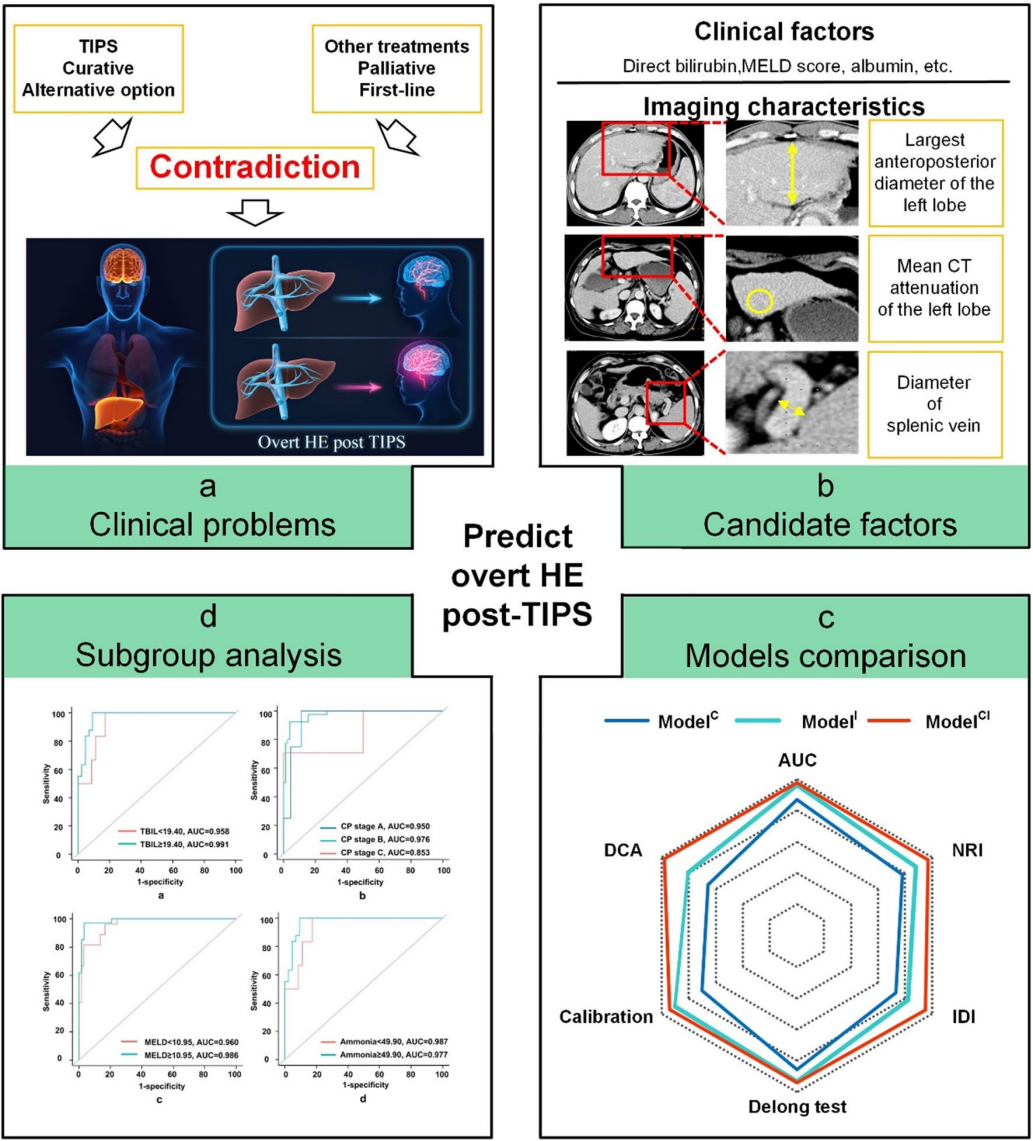

**Supplementary Information:**

The online version contains supplementary material available at 10.1007/s12072-021-10188-5.

## Introduction

Portal hypertension is a prevalent complication associated with liver cirrhosis and a common condition accompanying chronic liver diseases that may result in variceal bleeding and refractory ascites [1]. Currently, there are some effective treatments for these complications, such as endoscopic therapy, drug therapies (non-selective β-blockers with or without isosorbide mononitrate), large-volume paracentesis plus albumin, and transjugular intrahepatic portosystemic shunt (TIPS) [1, 2]. TIPS can establish artificial channels between the hepatic and portal veins to reduce the pressure in the portal vein [3]. Notably, rather than merely offering palliative effects, TIPS can provide a cure with minimal invasiveness that can significantly decrease and even normalise portal pressure, thereby simultaneously treating variceal bleeding and refractory ascites [2, 4]. However, compared to palliative treatments (such as large-volume paracentesis [5] and non-selective β-blockers [6]), the guidelines only recommend curative TIPS as an alternative option rather than first-line therapy for both variceal bleeding and refractory ascites [1, 7, 8]. One of the major reasons for this paradox is that hepatic encephalopathy (HE), particularly overt HE, may occur in up to 10% to 50% of patients within 1 year after TIPS [9]. The occurrence of overt HE after TIPS can negatively impact the quality of life and increase the mortality of patients [3, 10], thus impeding its wider application. Therefore, if we can preoperatively predict the risk of overt HE TIPS, then the decision to perform the TIPS procedure can be made more rationally and the TIPS can be appropriately applied to benefit more patients with symptomatic portal hypertension [11].

Several studies have preliminarily explored factors related to HE [12–17]. However, the following aspects require further exploration: (1) preoperative rather than postoperative factors should be used to ensure that the model can truly assist with preoperative patient selection for TIPS; (2) identified isolated risk factors should be integrated into combined models so that clinicians can calculate the quantitative score to predict the risk of overt HE post-TIPS; (3) the aforementioned models should be tested against a proper validation dataset to control overfitting problems and ensure the robustness of the model; and (4) considering the hemorrhage risk of cirrhosis, more noninvasive factors assessing the morphological changes of the liver [18, 19] such as imaging characteristics should be explored. Therefore, to truly identify patients suitable for TIPS [3], a combined model should be established to resolve the aforementioned challenges.

During our study based on a clinical database from two hospitals, we combined clinical factors and imaging characteristics to construct a noninvasive and integrated model with an effective validation dataset. Through this process, we hope to provide a reliable model for the preoperative prediction of the risk of overt HE post-TIPS to appropriately select patients for this curative TIPS.

## Materials and methods

### Patient selection

Patients treated with the TIPS between January 2013 and December 2018 were screened. Data were collected from Nanfang Hospital and Zhuhai People’s Hospital in China. All patients underwent TIPS treatment because of variceal rebleeding and/or refractory ascites. A total of 185 patients were included. The inclusion criteria were as follows: (1) at least one variceal rebleeding or refractory ascites after treatments such as vasoactive drugs, endoscopic treatment, or large-volume paracentesis; (2) bifurcation of the left and right branches of the portal vein was punctured from the right hepatic vein during TIPS therapy; (3) regular follow-up for at least 1 year.

The exclusion criteria were as follows: (1) TIPS performed to prevent failure or rebleeding after the initial pharmacological and endoscopic therapy (early TIPS); (2) age younger than 18 years; (3) pregnancy; (4) hepatocellular carcinoma that did not meet the Milano criteria for transplantation (i.e., a single lesion < 5 cm or fewer than three lesions with the largest measuring ≤ 3 cm); (5) creatinine level > 265 μmol/L; (6) Child–Pugh score > 13 points; (7) stents stenosis or occlusion during follow-up; (8) the portosystemic pressure gradient (PPG) did not meet the standard after TIPS treatment (PPG decreased > 50% from baseline or < 12 mmHg) [7, 9]; and (9) total portal vein thrombosis and severe medical comorbidities, such as septicaemia, extensive cardiovascular or cerebrovascular disease.

The study protocols were approved by the Ethics Review Committee of the Zhuhai People’s Hospital. Informed consent for medical research was waived because the patients’ data were collected retrospectively. All patients’ data were anonymised before analysis.

### Preoperative treatments

According to the guidelines, the following necessary preoperative treatments were performed: (1) anemia and coagulopathy were corrected to ensure patient safety during TIPS treatment (hemoglobin > 7 g/dL and prothrombin time < 25 s); (2) abdominal paracentesis was performed before TIPS to prevent massive hemorrhage; (3) vasoactive drugs (terlipressin [2 mg every 4 h], somatostatin [250 to 500 μg per h], or octreotide [25 to 50 μg per h]), and prophylactic antibiotics (ceftriaxone [1 g every 24 h]) were administered before TIPS [1]; (4) before TIPS, all patients underwent abdominal computed tomography to identify any variceal or spontaneous shunt that may lead to additional portal shunts and increase the risk of overt HE. If so, then embolisation of the abnormal shunts could be planned and performed using intraoperative angiography if necessary [3].

### TIPS procedures

All TIPS procedures were performed by three physicians, each of whom had more than 10 years of experience with interventional radiology. The TIPS procedure was performed as follows: (1) after general anesthesia, the bifurcation of the left and right branches of the portal vein was punctured from the right hepatic vein and the preoperative PPG was measured before stent deployment; (2) before stent implantation, we used a 6-mm balloon to expand the puncture channel and implanted an 8-mm polytetrafluoroethylene-covered stent; (3) to prevent stent dilation after TIPS, an 8-mm balloon was used to perform dilatation again to ensure the stent was expanded to 8 mm; (4) after stent insertion, portography was performed to enable visualisation of the left and right branches of the portal vein; and, (5) finally, we measured the postoperative PPG again. Patients with PPG reduction more than 50% from baseline or < 12 mmHg were identified as having achieved successful TIPS [3].

### Follow-up

According to the guidelines, all patients did not receive oral medicine (lactulose, rifaximin, etc.) after TIPS until HE occurred [9]. For the included patients, the baseline demographic characteristics and CT images were collected within 7 days before the TIPS procedure. All patients remained hospitalised after TIPS treatment until their conditions met the discharge criteria (e.g., normalisation of liver function and ammonia). Follow-up was performed once per week in the outpatient department for the first month; then, follow-up, including telephone interviews, outpatient visits, or hospital visits, was scheduled every 4 weeks. The patients and their families were asked to contact a physician immediately if any alteration in the patients’ mental state occurred.

The occurrences of HE, such as lethargy, apathy, and obvious personality changes, were recorded in detail. In this case, after repeated confirmation, the stage and degree of HE were evaluated. Grade II HE or higher according to the West Haven Criteria was considered overt HE. Patients were followed up until the end of the study (December 2019), liver transplantation, or death.

### Candidate factors

Clinical factors such as, besides factors listed in Table 1, we also included: the ratio of direct bilirubin and indirect bilirubin (DIR), thrombin time, and activated partial thromboplastin time were recorded.

**Table 1:** Baseline demographics of patients included in the study

	Training dataset (*N* = 130)	Validation dataset (*N* = 55)	*p* value
Age (year)	50.5 (18.00–78.00)	53.00 (26.00–77.00)	0.137
Sex (*N*)			0.564
Male	104	46	
Female	26	9	
Aetiology (*N*)			0.621
Alcohol	12	6	
Hepatitis B	95	36	
Hepatitis C	4	1	
Others	19	12	
CP score (point)	8.0 (5.0–12.0)	7.00 (5.0–11.0)	0.071
MELD score (point)	11.2 (6.4–19.4)	10.2 (6.4–22.4)	0.113
ALT (U/L)	20.0 (10.0–76.0)	20.0 (10.0–83.0)	0.575
AST (U/L)	32.0 (11.0–98.0)	29.0 (13.0–110.0)	0.310
DBIL (μmol/L)	9.1 (2.2–57.4)	10.0 (2.1–54.2)	0.689
IBIL (μmol/L)	9.9 (2.0–47.3)	10.0 (2.4–48.2)	0.516
Ammonia (μmol/L)	48.9 (8.0–156.0)	54.6 (27.4–185.5)	0.342
Albumin (g/L)	32.6 ± 5.1	32.6 ± 5.2	0.987
Serum sodium (mmol/L)	140.0 (108.9–152.0)	141.0 (133.0–155.0)	0.187
INR	1.3 (1.0–2.3)	1.2 (0.9–1.7)	0.339
Diabetes (*N*)			0.642
No	112	49	
Yes	18	6	
Liver cancer (*N*)			0.441
No	115	51	
Yes	15	4	

*CP* Child–Pugh, *ALT* alanine aminotransferase, *AST* aspartate aminotransferase, *DBIL* direct bilirubin, *IBIL* indirect bilirubin, *INR* international normalized ratio, *Liver cancer* accompanied by liver cancer

For imaging characteristics, considering that the morphologic changes of the liver observed using CT may reflect the severity of cirrhosis, which may be related to the risk of overt HE post-TIPS treatment, the following 18 imaging characteristics were measured: (1) maximum diameters of the hepatic fissure (Supplementary Fig. 1a), portal vein (Supplementary Fig. 1b), and splenic vein (Supplementary Fig. 1c); (2) number of depressions in the liver (depth ≥ 3 mm was defined as positive) (Supplementary Fig. 1d); (3) cavernous transformation of the portal vein (Supplementary Fig. 1e); (4) portal vein thrombosis (Supplementary Fig. 1f); (5) autologous shunt such as a gastro-renal shunt (Supplementary Fig. 1g), spleno-renal shunt (Supplementary Fig. 1h), or superficial epigastric vein shunt (Supplementary Fig. 1i); (6) anteroposterior (Supplementary Fig. 2a) or transverse (Supplementary Fig. 2b) maximum diameter ratio between the left vs. right lobe (measured in the slice of the middle hepatic vein); (7) anteroposterior (Supplementary Fig. 2c) or transverse (Supplementary Fig. 2d) diameter ratio between the left vs. right lobe (measured in the slice with largest diameter); (8) diameter ratio of portal vs. middle hepatic vein (Supplementary Fig. 2e) and portal vs. splenic vein (Supplementary Fig. 2f); (9) diameter ratio of hepatic fissure vs. liver transverse (Supplementary Fig. 2g) and hepatic fissure vs. liver anteroposterior (Supplementary Fig. 2h); and (10) mean CT attenuation ratio of left lobe vs. right lobe (Supplementary Fig. 2i).

### Outcome

The outcome of this study was overt HE post-TIPS, which was defined as grade II, grade III, and grade IV according to the West Haven Criteria [9]. Grade II is defined as the occurrence of lethargy or apathy, disorientation, obvious personality change, inappropriate behavior, dyspraxia, or asterixis. Grade III is defined as the occurrence of somnolence or semi-stupor, responsive to stimuli, confusion, gross disorientation, or abnormal behavior. Grade IV is defined as the occurrence of coma.

### Statistical analysis

Quantitative data are expressed as means (standard deviations) or medians (ranges) based on their distribution. Their distributions between groups were compared using the *t *test or Wilcoxon rank sum test, as appropriate. Similarly, categorical variables are displayed as percentages; they were compared using Pearson’s chi-squared test or Fisher’s exact test.

For model construction, after randomly dividing the cases into training and validation datasets, we used logistic regression to screen the clinical and imaging factors related to the risks of overt HE post-TIPS. Then, we used the identified clinical factors for our clinical model (Model^C^), the identified imaging factors for our imaging model (Model^I^), and all factors for our combined model (Model^CI^).

We first compared the discrimination of the three models using a receiver-operating characteristic (ROC) analysis involving the Delong test, net reclassification improvement (NRI), and integrated discrimination improvement (IDI). Then, we compared their calibration (by calibration plot) and decision curve analysis (DCA) results. Subsequently, we constructed a nomogram for the best model. Finally, we compared the ROC curves of the subgroups divided by the pre-TIPS total bilirubin (TBIL) level, Child–Pugh score, or model of end-stage liver disease (MELD) score to further test the stability of our model in different subgroups.

All statistical tests were two-sided and *p* < 0.05 was considered statistically significant. Data analyses was performed using R statistical packages 4.0.2 (2020-06-22).

## Results

### Study population and their baseline

**Fig. 1:**
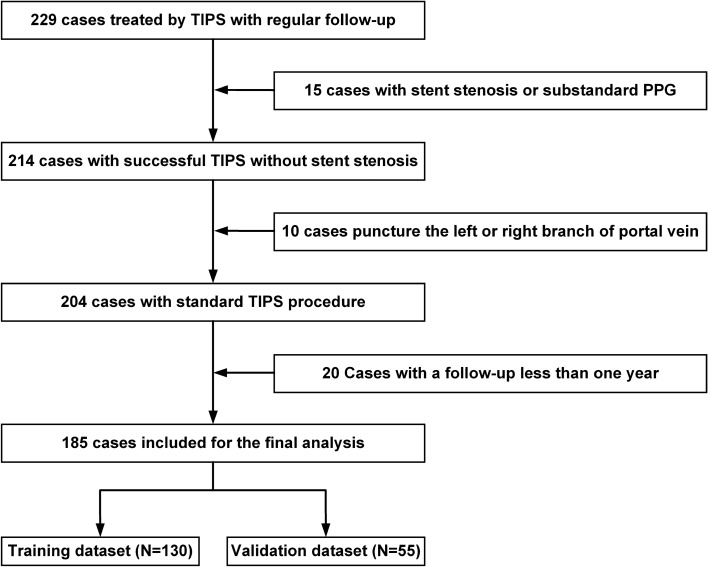
The inclusion and exclusion flowchart showing patient selection for this study. We screened 224 patients from two hospitals. After inclusion and exclusion criteria were evaluated, 185 patients were divided into the training dataset (130 cases) and validation dataset (55 cases)

A total of 185 patients were included in our study (Fig. 1) and randomised into the training dataset (130 participants) and validation dataset (55 participants). Symptoms leading to TIPS treatment included refractory ascites (24 patients: training dataset, 19 cases; validation dataset, 5 cases) and variceal bleeding (161 patients: training dataset, 111 cases; validation dataset, 50 cases). There were no statistical differences in the demographic factors of the training and validation datasets. Baseline characteristics of the patients are reported in Table 1.

### Construction of models

Among all the clinical factors (such as age, sex, Child–Pugh score, MELD score) and imaging characteristics, after univariate and multivariate regression analyses, the direct bilirubin (DBIL), Child–Pugh score, hepatic fissure maximum diameter (HFMD), and diameter ratio of the portal vs. splenic vein (PSR) were statistically related to overt HE after TIPS (Supplementary Tables 1, 2).

Based on these results, we constructed a clinical model (Model^C^), including the DBIL and Child–Pugh score, an imaging model (Model^I^), including HFMD and PSR, and a combined model (Model^CI^), including all four factors identified by multivariate regression (Table 2).

**Table 2:** Multivariate logistic regression analysis

Factors	OR (95% CI)	*p* value
DBIL	0.842 (0.706–0.960)	0.032
Child–Pugh score	3.205 (1.748–6.977)	< 0.001
HFMD	3.293 (2.110–6.108)	< 0.001
PSR	13.008 (1.012–293.831)	0.072

*OR* odds ratio, *CI* confidence interval, *DBIL* direct bilirubin, *HFMD* hepatic fissure maximum diameter, *PSR* diameter ratio of portal vs. splenic vein

### Comparison of models

**Fig. 2:**
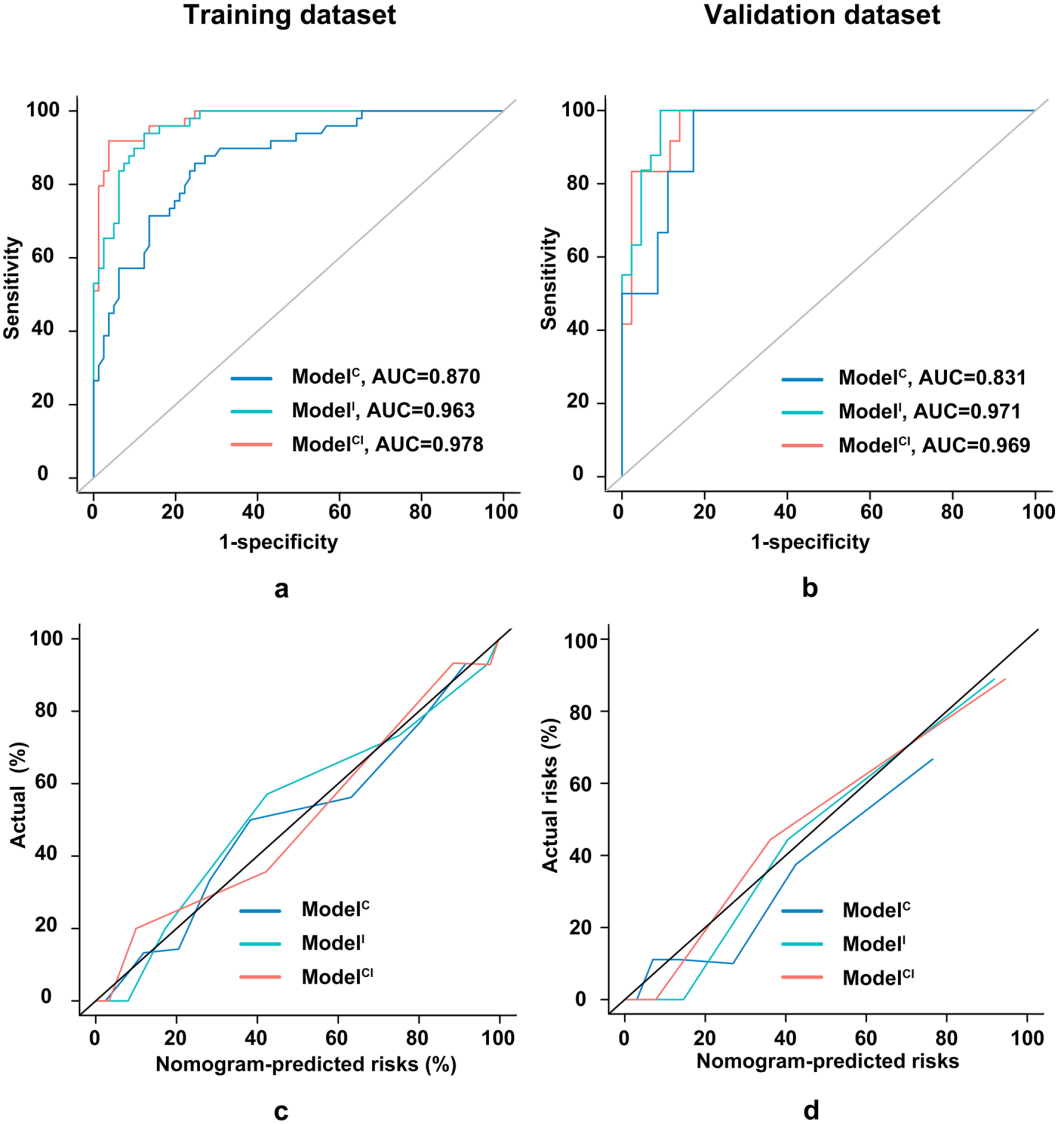
Model comparisons and optimal model identification. To predict overt HE post-TIPS, the AUCs of the clinical, imaging, and combined models were 0.870, 0.963, and 0.978 for the training dataset (**a**) and 0.831, 0.971, and 0.969 for the validation dataset (**b**). Calibrations are displayed for the training dataset (**c**) and validation dataset (**d**)

**Fig. 3:**
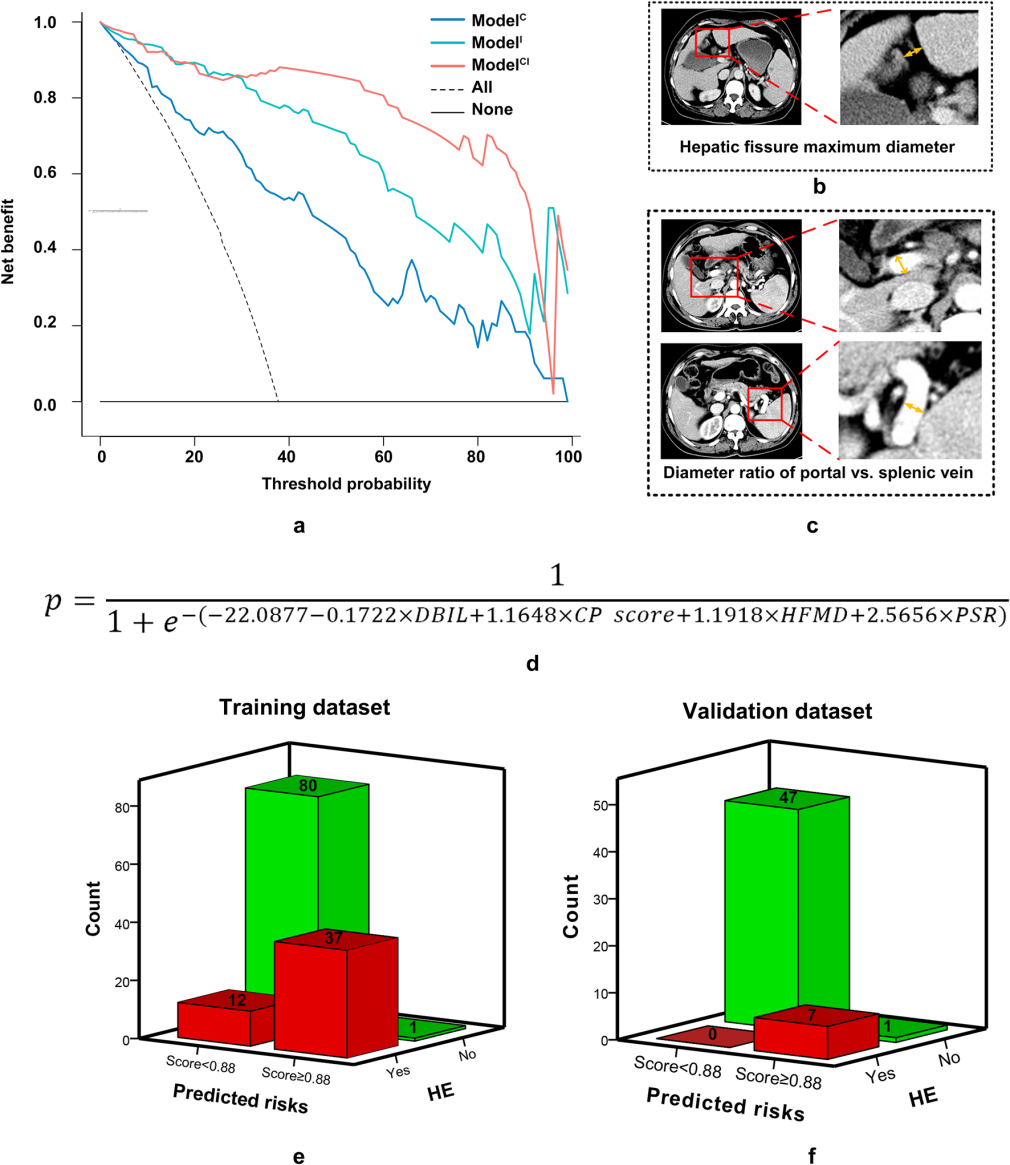
Imaging characteristics, decision curves, and equations of the combined model. The decision analysis curve of the three models were displayed (**a**). The combined model (Model^CI^) included two clinical factors (DBIL and CP score) and two imaging characteristics: hepatic fissure maximum diameter (**b**) and diameter ratio of portal vs. splenic vein (**c**). Its equation is displayed (**d**). When divided by the cut-off value of Model^CI^ (score of 0.88), the two subgroups had significantly statistically differences (both *p* < 0.001) in the training (**e**) and validation (**f**) datasets. HFMD: hepatic fissure maximum diameter; PSR: diameter ratio of portal vs. splenic vein

We compared the three models based on discrimination, calibration, and decision curves. For discrimination, the areas under the curve (AUCs) of Model^C^, Model^I^, and Model^CI^ were 0.870, 0.963, and 0.978, respectively, for the training dataset and 0.831, 0.971, and 0.969, respectively, for the validation dataset (Fig. 2a, b). Model^CI^ and Model^I^ were superior to Model^C^ (according to the Delong test, NRI, and IDI), and Model^CI^ performed better than Model^I^ in the training dataset (Supplementary Table 2). Regarding calibration, Model^CI^ was comparable to Model^I^ but superior to Model^C^ (Fig. 2c, d). Regarding the decision curve, Model^CI^ also performed better than Model^C^ and Model^I^ (Fig. 3a). Based on these results, Model^CI^ was chosen as the final model (Fig. 3b, c, d).

### Subgroup analysis

Based on the AUC of Model^CI^, a cut-off value of 0.88 with the best Youden index was identified. The proportion of overt HE was significantly statistically different between the low-risk subgroup (Model^CI^ score < 0.88) and high-risk subgroup (Model^CI^ score ≥ 0.88) in the training dataset (13.3% vs. 97.4%; *p* < 0.001) (Fig. 3e) and validation dataset (0.0% vs. 87.5%; *p* < 0.001) (Fig. 3f).

**Fig. 4:**
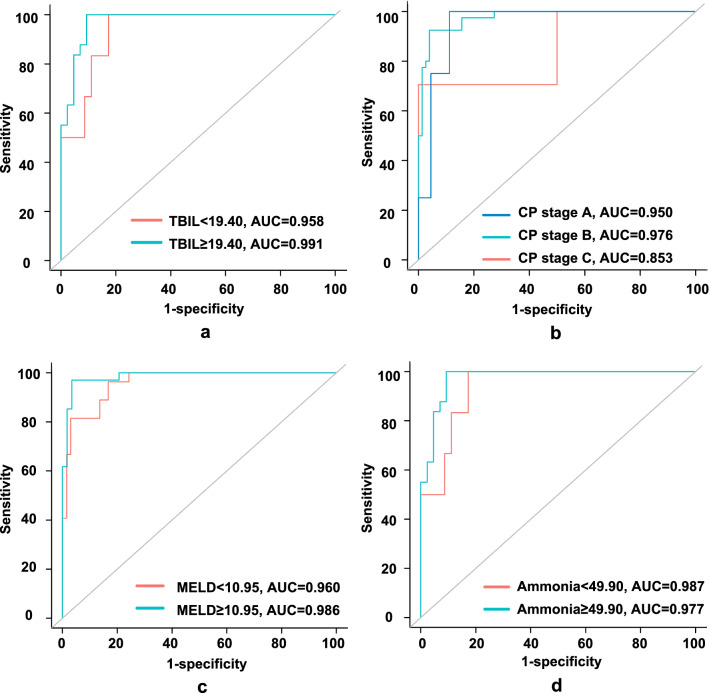
Subgroup analysis of Model^CI^. After dividing by the preoperative median TBIL (**a**), Child–Pugh stage (**b**), median MELD score (**c**), and median ammonia level (**d**), the AUCs showed no statistical differences

We performed tests to determine whether the pre-TIPS TBIL, Child–Pugh stage, and MELD score influenced performance. The results showed that the discrimination between subgroups was not statistically different (Supplementary Table 3): TBIL < 19.40 vs. TBIL ≥ 19.40 (0.958 vs. 0.991) (Fig. 4a); Child–Pugh stage A vs. stage B vs. stage C (0.950 vs. 0.976 vs. 0.853) (Fig. 4b); MELD score < 10.95 vs. MELD score ≥ 10.95 (0.960 vs. 0.986) (Fig. 4c); preoperative ammonia < 49.90 vs. preoperative ammonia ≥ 49.90 (0.987 vs. 0.977) (Fig. 4d).

## Discussion

During our study, we constructed a noninvasive integrated model to preoperatively predict overt HE post-TIPS treatment. Our model satisfied discrimination and calibration in both the training and validation datasets. During the subgroup analysis, its performance was not affected by the TBIL, MELD score, Child–Pugh score, or preoperative ammonia level. Based on these results, our model could assist with appropriately selecting patients to undergo TIPS treatment, thereby reducing the incidence of overt HE post-TIPS, which would make the decision to perform TIPS treatment more rational and scientifically based.

Among the current treatments used for portal hypertension in cirrhosis, TIPS is the only minimally invasive method that can decrease the portal pressure [4] and simultaneous treat variceal bleeding and refractory ascites [20, 21]. However, it has not been recommended as the first-line therapy [1, 7, 8]. One of the major reasons for this contradictory finding is that TIPS treatment may cause an increase in some toxic substances in the central nervous system, thereby leading to post-TIPS HE in 25% to 50% of patients with cirrhosis [4, 22, 23]. HE, particularly overt HE, has a significant negative effect on the quality of life and survival rate of patients [24]. Therefore, quantitively predicting the risk of overt HE can provide crucial information that can be used to guide the decision to perform TIPS.

To address this issue, we combined traditional clinical factors and imaging characteristics designed to assess the severity of cirrhosis and portal hypertension. Regarding clinical factors, the preoperative DBIL and Child–Pugh score were related to overt HE post-TIPS. It was noticeable that DBIL rather than TBIL and indirect bilirubin (IBIL) was more informative of the risk of overt HE post-TIPS. One explanation for this is that high direct bilirubin levels are related to hepatocellular dysfunction and observed neuronal toxicity, and they are significantly related to HE [25, 26]. The Child–Pugh score, another frequently identified risk factor for HE, rather than the MELD score, was used in the final model. This could be because the MELD score had limited impact on the emotional state [27]. To confirm that our model could be used for preoperative treatment decision-making, intraoperative and postoperative factors (such as decrease in PPG and hepatic venous pressure gradient) were not included. However, whether they could provide additional information regarding treatment after the TIPS procedure requires further exploration.

Regarding imaging characteristics, an increase in the maximum diameter of the liver fissure and shrinkage of the liver caused by cirrhosis were more obvious and indicated that the detoxification function and compensatory capacity of the liver had been decreased [28]. However, the increased ratio of the diameters of the portal and splenic veins showed that more blood from the superior mesenteric vein flowed into the portal vein before TIPS. Because toxic substances (especially plasma ammonia) in the intestinal system were mainly absorbed in the superior mesenteric vein after TIPS, more undetoxified portal vein blood would flow directly into the nervous system through the shunt vessel, thereby bringing more ammonia into the brain [29].

Using our constructed models, we observed that the AUC of Model^C^ was 0.831 for the validation dataset, which was comparable to those reported by previous studies (between 0.743 and 0.872) [12, 22, 30]. However, the AUCs of Model^I^ were significantly higher (0.963 and 0.971 for the training dataset and validation dataset, respectively), the performances of NRI, IDI, and the Delong test were statistically superior, and there were improvements in the calibration and DCA analysis results. These results revealed the importance of including imaging characteristics to predict overt HE post-TIPS. We observed that Model^CI^ outperformed Model^I^ in the NRI, IDI, and DCA analyses. These results demonstrated that clinical factors contributed to improvements in the model. Based on these results, both clinical and imaging factors were indispensable for predicting overt HE post-TIPS.

This study had some limitations. First, the small sample size did not allow for a more detailed analysis of, for instance: (1) whether the time-dependent risks of overt HE post-TIPS could be calculated; (2) the Child–Pugh score included two subjective criteria (ascites and HE), it could not be determined whether it could truly outperform the MELD score. Second, to control the possible confounding factors, patients with a decrease in the PPG less than 50% or 12 mmHg or stent stenosis were excluded. Therefore, whether our conclusion is applicable to these patients requires further exploration. Third, there were differences between Eastern and Western patients (e.g., viral cirrhosis and alcoholic cirrhosis); therefore, validation of our results for a Western cohort should be considered for future studies. For example, studies considering the advantages of smaller stents and small body frames of Chinese patients [31] should be performed because the participating hospitals used only 8-mm-diameter stents for the TIPS procedure. Because HE has potentially different influences with 8-mm and 10-mm stents [9], whether our conclusions are applicable to those treated with 10-mm-diameter stents need further validation. Fourth, although the AUCs of Model^CI^ in our study were as high as 0.969 in the validation dataset, whether some potential factors such as assessments of sarcopenia, preoperative hepatic venous pressure gradient and novel biomarkers could further improve the performance requires further evaluation [14, 15, 32]. Finally, because of the difficulty assessing minimal HE using retrospective data, this study only analysed overt HE; however, minimal HE data should be included in future studies.

In conclusion, our individualised model can predict overt HE post-TIPS treatment; therefore, it can assist with treatment decisions. For low-risk populations (such as patients with a risk score < 0.88), TIPS can be performed safely and may be considered as first-line therapy. Conversely, for high-risk populations (such as patients with a risk score ≥ 0.88), TIPS may be performed more prudently when inevitable. Furthermore, more preventive treatments and closer follow-up after TIPS treatment should also be considered.

## Supplementary Information

Below is the link to the electronic supplementary material.

Supplementary file1 (DOCX 1492 kb)

## Data Availability

Due to the privacy of patients, the data related to patients cannot be available for public access but can be obtained from the corresponding author on reasonable request approved by the institutional review board of Zhuhai People’s Hospital (llg0902@sina.com).
