# Supplementary material for: Prediction of overt hepatic encephalopathy after transjugular intrahepatic portosystemic shunt treatment: a cohort study
Source: Hepatol Int. 2021 May 11;15(3):730–40. doi: 10.1007/s12072-021-10188-5 (PMC8286937; doi:10.1007/s12072-021-10188-5)
Supplement: Supplementary file 1 — Supplementary file1 (DOCX 1492 kb) [file 12072_2021_10188_MOESM1_ESM.docx]

**Prediction of overt hepatic encephalopathy after transjugular intrahepatic portosystemic shunt treatment: a cohort study**

**Authors**

Yang Yang^1*^, M.D; Sirui Fu^1*^, M.D; Bin Cao^2*^, M.D; Kenan Hao^3*^, M.D; Yong Li^1^, M.D; Jianwen Huang^1^, M.D; Wenfeng Shi^4^, Bachelor; Chongyang Duan^5^, Ph.D; Xiao Bai^1^, M.D; Kai Tang^1^, M.D; Shirui Yang^6^, M.D; Xiaofeng He^3#^, M.D; Ligong Lu^1#^, M.D;

**Supplementary Material**

**Table of Contents**

**Supplementary Fig. 1**

**Supplementary Fig. 2**

**Supplementary Table 1**

**Supplementary Table 2**

**Supplementary Table 3**

**
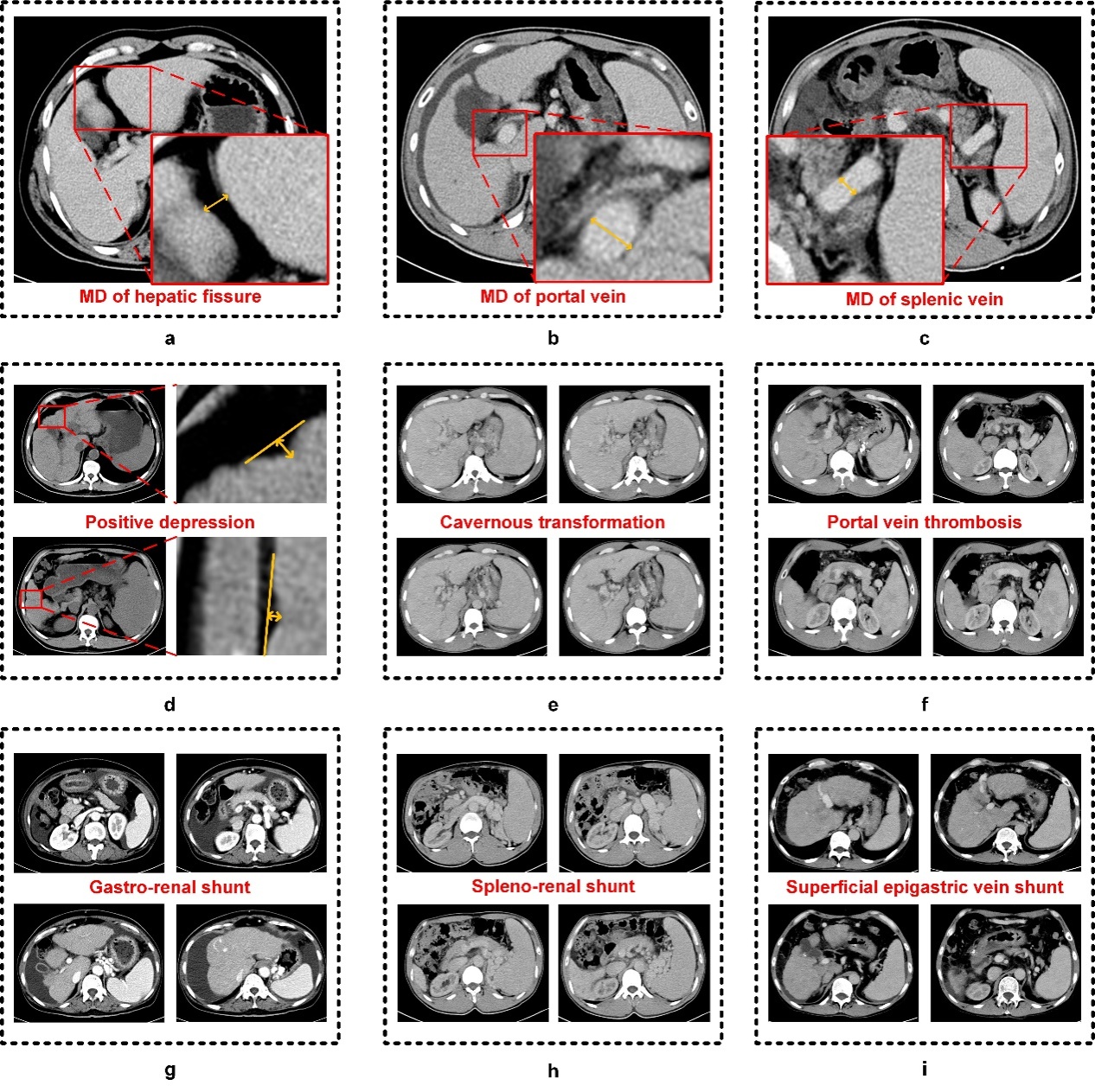
**

**Supplementary Fig. 1. Imaging characteristics: part I.** Maximum diameters of the hepatic fissure (a), portal vein (b), and splenic vein (c). The number of depressions in the liver: a depth ≥ 3 mm was defined as positive (d). Cavernous transformation of the portal vein (e), portal vein thrombosis (f), gastro-renal shunt (g), spleno-renal shunt (h), and superficial epigastric vein shunt (i).

**
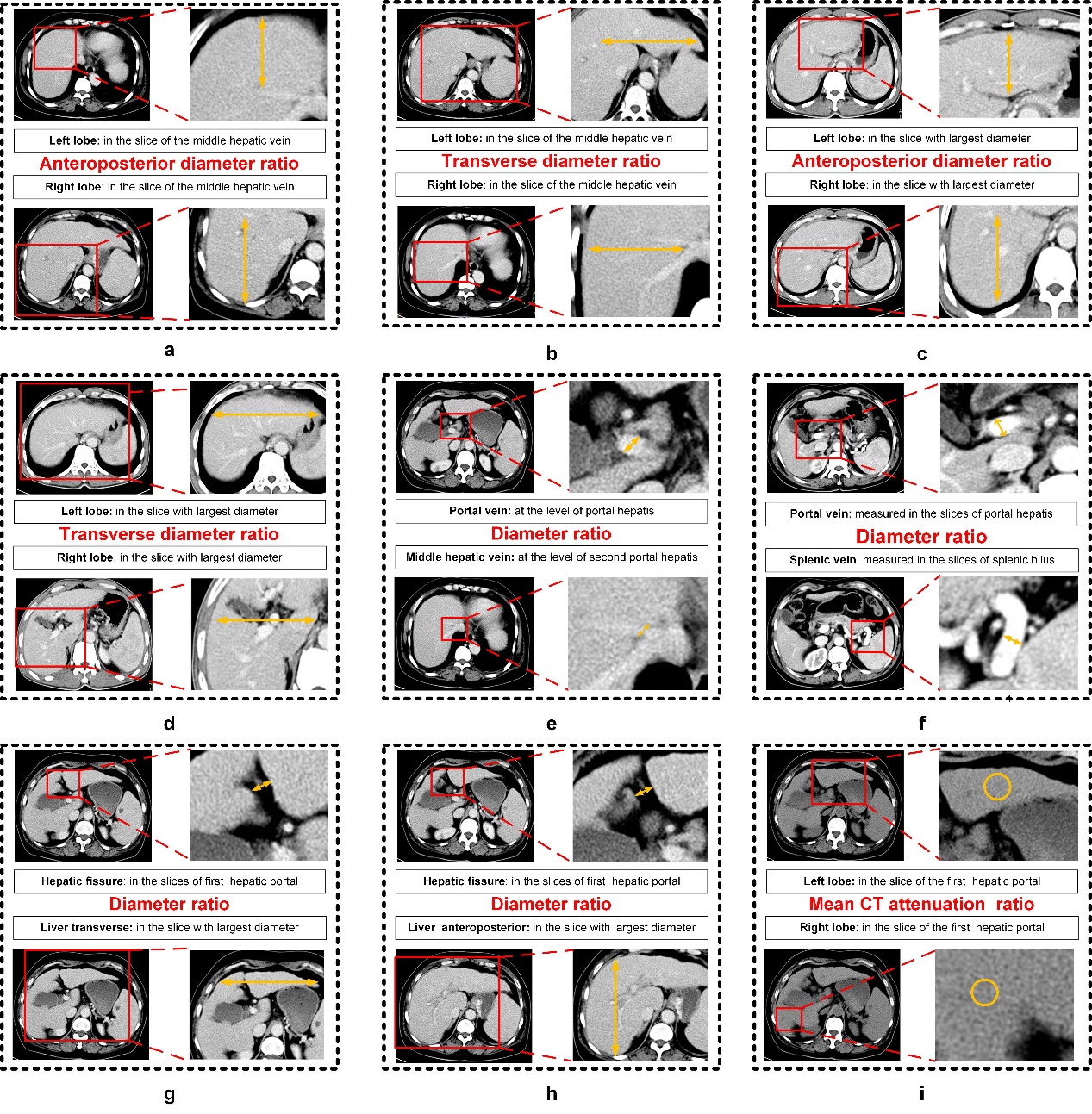
**

**Supplementary Fig. 2. Imaging characteristics: part II.** Anteroposterior (a) and transverse (b) MDRLR (measured in the slice of the middle hepatic vein). Anteroposterior (c) and transverse (d) MDRLR (measured in the slice of the maximum diameter). Ratio of the diameters of the portal and middle hepatic veins (e). Ratio of the diameters of the portal and splenic veins (f). Ratio of the diameters of the hepatic fissure and liver transverse (g). Ratios of the diameters of the hepatic fissure and anteroposterior maximum diameter of the liver (h). Mean ratio of attenuation of the left lobe and right lobe on CT (i). MDRLR: ratio of the maximum diameters of the left and right lobe.

| **Supplementary Table 1. Univariate logistic regression analysis** | | |
| --- | --- | --- |
| **Factors** | **OR (95% CI)** | ***p*-value** |
| **Clinical factors** |  |  |
| Age | 1.016 (0.983–1.050) | 0.344 |
| Sex | 0.644 (0.270–1.537) | 0.322 |
| Etiology |  | 0.667 |
| Alcohol |  | Reference |
| Hepatitis B | 0.722 (0.142–3.670) | 0.695 |
| Hepatitis C | 1.444 (0.505–4.131) | 0.493 |
| Others | 2.167 (0.244–19.276) | 0.488 |
| Reasons for TIPS | 1.043 (0.381–2.859) | 0.934 |
| Child-Pugh score | 2.460 (1.744–3.470) | <0.001^#^ |
| MELD score | 1.092 (1.004–1.189) | 0.041^#^ |
| ALT | 1.000 (0.974–1.026) | 0.988 |
| AST | 1.021 (1.000–1.042) | 0.051^#^ |
| DBIL | 0.989 (0.952–0.997) | 0.090^#^ |
| IBIL | 0.998 (0.949–1.050) | 0.948 |
| DBIL/IBIL ratio | 1.082 (0.609–1.924) | 0.787 |
| Albumin | 0.903 (0.838–0.974) | 0.008^#^ |
| INR | 12.401 (1.863–82.573) | 0.009^#^ |
| TT | 1.039 (0.898–1.203) | 0.606 |
| APTT | 1.039 (1.004–1.076) | 0.030^#^ |
| Ammonia | 1.014 (1.001–1.027) | 0.034^#^ |
| Creatinine | 1.004 (0.993–1.015) | 0.476 |
| Serum sodium | 0.906 (0.830–0.988) | 0.026^#^ |
| Liver cancer accompany | 1.768 (0.530–5.894) | 0.354 |
| Diabetes | 2.351 (0.727–7.602) | 0.153 |
| **Radiological characteristics** |  |  |
| MD of hepatic fissure | 3.779 (2.349–6.078) | <0.001^#^ |
| MD of portal vein | 1.051 (0.907–1.219) | 0.507 |
| MD of splenic vein | 0.843 (0.744–0.956) | 0.008^#^ |
| Positive depression | 1.010 (0.982–1.039) | 0.505 |
| Cavernous transformation | 0.584 (0.139–2.452) | 0.463 |
| Portal vein thrombosis | 0.750 (0.260–2.162) | 0.594 |
| Gastro-renal/spleno-renal shunt | 0.603 (0.281–1.295) | 0.603 |
| Superficial epigastric vein shunt | 1.267 (0.606–2.648) | 0.530 |
| Anteroposterior diameter ratio between LvR in the slice of MHV | 0.459 (0.078–2.698) | 0.389 |
| Transverse diameter ratio between LvR in the slice of MHV | 0. 554 (0.172–1.782) | 0.322 |
| Anteroposterior diameter ratio between LvR in the slice of MD | 1.103 (0.186–6.532) | 0.914 |
| Transverse diameter ratio between LvR in the slice of MD | 0.363 (0.093–1.414) | 0.144 |
| Diameter ratio of portal vs middle hepatic vein | 1.044 (0.781–1.395) | 0.774 |
| Diameter ratio of portal vs. splenic vein | 16.180 (4.048–64.676) | <0.001^#^ |
| Diameter radio of hepatic fissure vs. liver transverse | 1.453 (0.180–11.746) | 0.726 |
| Diameter radio of hepatic fissure vs. liver Anteroposterior | 4.691×10^65^(3.337×10^42^–6.593×10^88^) | <0.001^#^ |
| Mean CT attenuation between LvR of the first hepatic vein | 0.087 (0.002–4.792) | 0.233 |
| ^#^Factors with *p*<0.100 were used in the multivariate analysis  ALT: alanine aminotransferase; APTT: activated partial thromboplastin time; AST: aspartate aminotransferase; DBIL: direct bilirubin; IBIL: indirect bilirubin; INR: international normalized ratio; LvR: left vs. right lobe; MD: maximum diameter; MELD: model of end-stage liver disease; MHV: middle hepatic vein; OR: odds ratio; TT: thrombin time. | | |

| **Supplementary Table 2. Pairwise comparisons of models** | | | |
| --- | --- | --- | --- |
|  | **NRI** | **IDI** | **Delong test** |
| **Training dataset** |  |  |  |
| Model^C^ vs. Model^I^ | <0.001* | <0.001* | 0.003* |
| Model^I^ vs. Model^CI^ | 0.002* | <0.001* | 0.121 |
| Model^C^ vs. Model^CI^ | <0.001* | <0.001* | <0.001* |
| **Validation dataset** |  |  |  |
| Model^C^ vs. Model^I^ | 0.001* | 0.001* | 0.029* |
| Model^I^ vs. Model^CI^ | 0.124 | 0.509 | 0.919 |
| Model^C^ vs. Model^CI^ | <0.001* | <0.001* | 0.010* |
| Data are presented as *p* values.  IDI: integrated discrimination improvement; NRI: net reclassification improvement *Statistically significant differences | | | |

| **Supplementary Table 3. Subgroup analysis of AUCs** | | |
| --- | --- | --- |
| **Subgroups divided by** | **Z** | ***P* value** |
| **TBIL** (<19.40 vs. ≥19.40) | -0.874 | 0.382 |
| **Child-Pugh stage** |  |  |
| A vs B | -0.423 | 0.672 |
| A vs C | 0.413 | 0.680 |
| B vs C | 0.538 | 0.590 |
| **MELD score** (<7.94 vs. ≥7.94) | -0.627 | 0.531 |
| **Ammonia** (<49.90 vs. ≥49.90) | 0.356 | 0.722 |
